# 

**Published:** 1897-06-12

**Authors:** 


### EARLY EDITION.

**Notices of Births, Deaths, and Marriages** are inserted in this column at a charge of 2s. 6d. for an announcement not exceeding four lines.

### NOTICE TO CORRESPONDENTS.

All MS., Letters, Books for Review, and other matters intended for the Editor should be addressed The Editor, "The Hospital," The Hospital Building, 28 & 29, Southampton Street, Strand, W.C.

The Editor cannot undertake to return rejected MS., even when accompanied by stamped directed envelope.

**Notice to Advertisers and Subscribers.**

All Advertisements, Orders for Copies of The Hospital, and Business Communications should be addressed to **THE MANAGER, (Not to The Editor),** The Hospital, The Hospital Building, 28 & 29, Southampton Street, Strand, London, W.C.

The Cost of Subscription, post free, is as follows:—Per week, 2½d.; per quarter, 2s. 8d.; per half-year, 5s. 3d.; per annum, 10s. 6d. Foreign:—Per annum, 15s. 2d.; payable, in all cases, in advance.

### BOOKS RECEIVED.

Bailliere, Tindall, and Cox.

"From Our Dead Selves to Higher Things." By James Gant, F.R.C.S.

"Excretory Irritation." By David Walsh, M.D.

The Cotton Press.

"Molière and his Medical Associations." By A. M. Brown, M.D.

Sampson Low, Marston, and Co.

"Left-Hand Writing." By John Jackson, F.E.I.S.

Kegan Paul, Trench, Trubner, and Co.

"Bradshaw's Dictionary of Mineral Waters, Climatic Health Resorts, &c." 1897.

Simpkin, Marshall, Hamilton, Kent, and Co.

"Thoughts for Mothers." With a Preface by the Lord Bishop of Lichfield.

Morton and Burt.

"Annual Report of the British Medical Benevolent Fund for 1896."

W. H. and L. Collingridge.

"A Contribution to the Natural History of Scarlet Fever." By John T. Wilson, M.D., D.P.H.

Reformatory and Refuge Union.

"A Classified List of Child-Saving Institutions."

Robert Anderson.

"Annual Report on the Sanitary Condition of the County of Lanark." By John T. Wilson, M.D.

